# Make Haste Slowly

**Published:** 1870-06

**Authors:** 


### *Make Haste Slowly.*

We give all due credit to the uneasy gentlemen who think they are engaged in the cheerful business of elevating the profession by loud declamation of their desires. But prior to adoption of the methods they wish to see in operation, we commend to them a remark of Lord Bacon: "Neither do I think ourselves yet learned or wise enough to wish reasonably; for as it asks some knowledge to demand a question not impertinent, so it asks some sense to make a wish not absurd."

### *Acknowledgment.*

Thanks are due to our friend, Thomas A. Elder, M. D., of Mifflintown, Penn., for early copies of papers containing the proceedings of the American Medical Association.

